# Geospatial pattern of level of minimum acceptable diet and its determinants among children aged 6–23 months in Ethiopia. Spatial and multiscale geographically weighted regression analysis

**DOI:** 10.3389/fpubh.2024.1348755

**Published:** 2024-06-19

**Authors:** Tegene Atamenta Kitaw, Biruk Beletew Abate, Befkad Derese Tilahun, Ribka Nigatu Haile

**Affiliations:** Department of Nursing, College of Health Science, Woldia University, Woldia, Ethiopia

**Keywords:** geospatial pattern, minimum acceptable diet, determinants, children, Ethiopia

## Abstract

**Background:**

Despite prior progress and the proven benefits of optimal feeding practices, improving child dietary intake in developing countries like Ethiopia remains challenging. In Ethiopia, over 89% of children fail to meet the minimum acceptable diet. Understanding the geographical disparity and determinants of minimum acceptable diet can enhance child feeding practices, promoting optimal child growth.

**Methods:**

Spatial and multiscale geographically weighted regression analysis was conducted among 1,427 weighted sample children aged 6–23 months. ArcGIS Pro and SatScan version 9.6 were used to map the visual presentation of geographical distribution failed to achieve the minimum acceptable diet. A multiscale geographically weighted regression analysis was done to identify significant determinants of level of minimum acceptable diet. The statistical significance was declared at *P*-value <0.05.

**Results:**

Overall, 89.56% (95CI: 87.85–91.10%) of children aged 6–23 months failed to achieve the recommended minimum acceptable diet. Significant spatial clustering was detected in the Somali, Afar regions, and northwestern Ethiopia. Children living in primary clusters were 3.6 times more likely to be unable to achieve the minimum acceptable diet (RR = 3.61, LLR =13.49, *p* < 0.001). Mother’s with no formal education (Mean = 0.043, *p*-value = 0.000), family size above five (Mean = 0.076, *p*-value = 0.005), No media access (Mean = 0.059, *p*-value = 0.030), home delivery (Mean = 0.078, *p*-value = 0.002), and no postnatal checkup (Mean = 0.131, *p*-value = 0.000) were found to be spatially significant determinants of Inadequate minimum acceptable diet.

**Conclusion:**

Level of minimum acceptable diet among children in Ethiopia varies geographically. Therefore, to improve child feeding practices in Ethiopia, it is highly recommended to deploy additional resources to high-need areas and implement programs that enhance women’s education, maternal healthcare access, family planning, and media engagement.

## Introduction

Sufficient nutrition is predominant for optimal physical and cognitive development of the child. Children’s morbidity and mortality associated with undernutrition are high (1). Minimum acceptable diet (MAD) is a crucial indicator for evaluating infant and young child feeding (IYCF) practices among children aged 6 to 23 months. It encompasses two essential components: minimum dietary diversity (MDD) and minimum meal frequency (MMF) (2). MDD ensures that a child’s diet includes a variety of food groups from different nutritional categories, while MMF ensures that the child receives an adequate number of meals and snacks throughout the day (3).

Maintaining a MAD during infancy and early childhood is crucial for ensuring optimal growth and development (4). Failure to achieve MAD places young children at heightened risk of undernutrition, particularly stunting and micronutrient deficiencies. These nutritional inadequacies can lead to increased morbidity and mortality (5). Underscoring the critical importance of providing adequate and balanced diets during this critical period of life.

Undernutrition, a state of inadequate nutrient intake, is a significant contributor to child mortality, claiming the lives of nearly half of all children under the age of five (6). A substantial proportion of childhood fatalities, amounting to almost one-third, occur in the African Region and are directly attributable to malnutrition (7).

In Ethiopia, a significant proportion of child mortality, amounting to 28%, is linked to inadequate nutrition (8). Only 4% of children between the ages of 6 and 23 months receive a diverse diet encompassing four or more food groups. Moreover, the frequency of feeding sessions is also suboptimal, with only 45% of children in this age group being fed at least thrice daily (9). This alarming situation is reflected in the low prevalence of optimal feeding practices among young children.

Previous research identified various factors influencing the likelihood of children not achieving a minimum acceptable diet (MAD). These factors include residential location (10), maternal age (11), maternal education level (12), socioeconomic status (13), exposure to media messaging about healthy eating (14), maternal employment status (15), place of delivery (16), frequency of antenatal care attendance (17), and participation in postnatal care services (18).

The Sustainable Development Goals (SDGs) have targeted reducing newborn and under-five mortality by 2030, aiming for as low as 12 and 25 deaths per 1,000 live births, respectively (19). Achieving these targets hinges significantly on ensuring that children consume a minimum acceptable diet (MAD). In line with this global initiative, Ethiopia’s Ministry of Health has also adopted a comprehensive plan to address malnutrition and diet-related public health issues, recognizing their crucial role in reducing under-five mortality. The ministry’s strategy promotes MAD among children as a critical intervention for achieving this goal (20).

Despite the enormous progress made previously and the known health benefits of optimal feeding practices, increasing the number of children getting a minimum acceptable diet in developing countries like Ethiopia has become a significant challenge. Thus, recent data regarding minimum acceptable is crucial for public health practitioners and policymakers in supporting efforts toward strengthening child nutrition programs and ending all types of malnutrition in the late life of children.

Most studies on minimum acceptable diet (MAD) in Ethiopia are confined to specific districts or regions. Additionally, logistic regression is the predominant method employed to identify factors influencing MAD. Logistic regression might provide information in situations where there is no variation across the study area. However, in the field of public health, such assumptions are not always valid, particularly when it is known that variables vary across the study area (i.e., spatially non-stationary). In such cases, logistic regression becomes inadequate for providing suitable information. Thus, geographical weighted regression is better suited for addressing these challenges.

According to previous reports, approximately 89% of children in Ethiopia failed to meet MAD requirements (21). However, the geospatial distribution of MAD among children in Ethiopia remains poorly understood, hindering the development of geographically targeted interventions. Furthermore, data on hot and cold spot areas for MAD is scarce. To expedite progress toward improving child feeding practices and ending child malnutrition, understanding the geospatial distribution of MAD and its determinants among children is crucial. Thus, this study aims to better understand the spatial pattern of the level of minimum acceptable diet and its determinants among children aged 6–23 months in Ethiopia.

## Methods

### Study setting, study period and data source

According to forecasts from trading economics and data from recent census figures, the total population of Ethiopia was 115.0 million by 2020 (22). The EDHS 2019 final report includes data at the country level from the nine regional states and two municipal administrations. The administrative levels were divided into zones, woreda, and so forth. A multilevel analysis was conducted among children between 6 to 23 months. The EDHS collects pertinent information mainly regarding maternity health care utilization, marriage and sexual behavior, child feeding practice, children and women’s dietary condition, and children’s and adult mortality. Data collection was carried out from March to June 2019 (23).

### Data extraction, population

First, the project proposal was sent to the Demographic and Health Surveys (DHS) Program. After a detailed review process, the DHS program accepted the proposal and granted access with an approval letter to use the survey datasets. Data extraction was done to select children aged 6–23 months. The data extraction was conducted between August 3 and 30, 2023. All children aged 6–23 months were the source population, whereas all children aged 6–23 months in the selected enumeration area were the study population.

### Sampling methods

**Figure 1:**
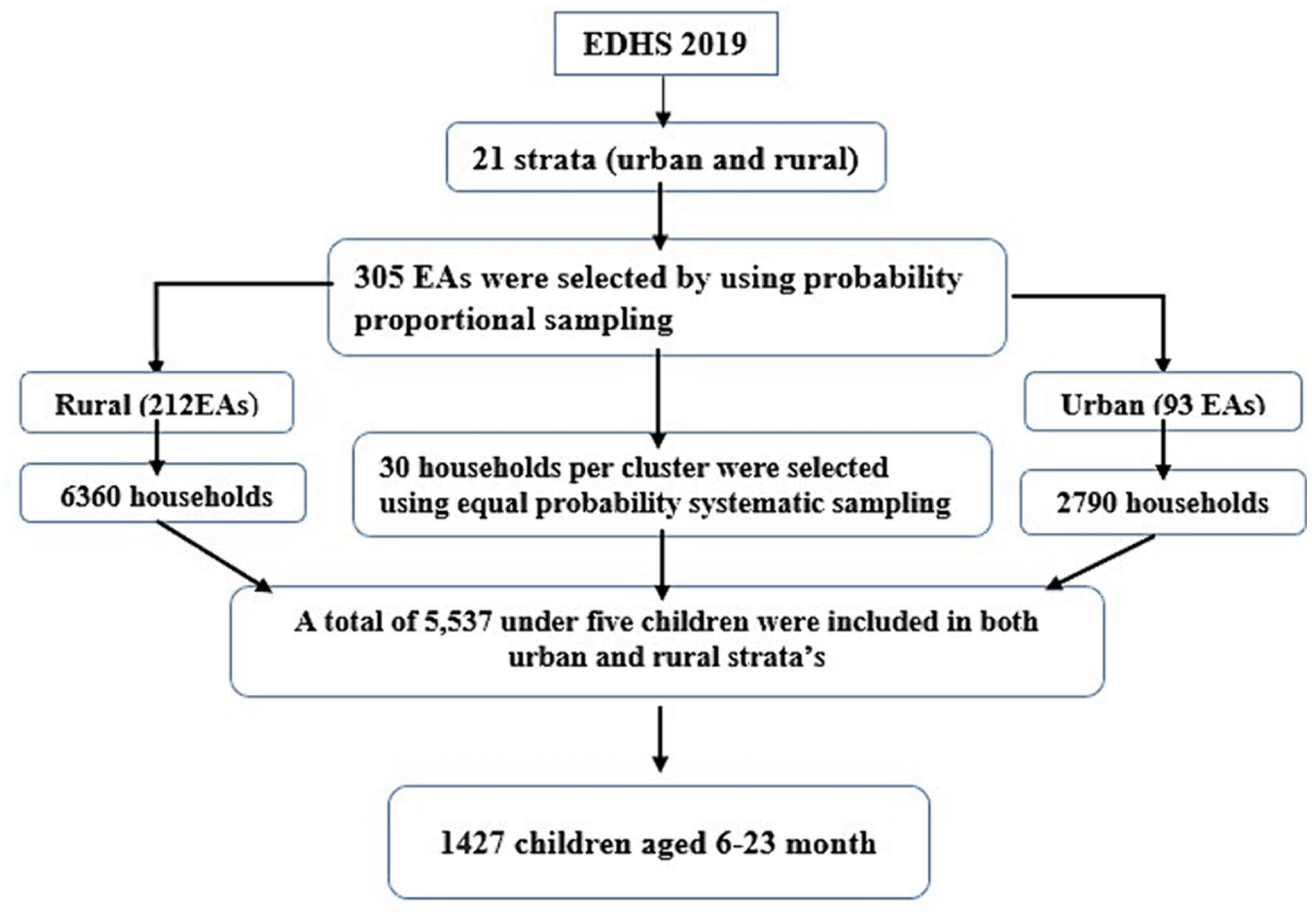
Schematic representation of the sampling procedures in the study of geospatial pattern of level of minimum acceptable diet and its determinants among children aged 6–23 months in Ethiopia, EDHS 2019 N.B EAs, enumeration areas.

The EDHS 2019 sample was stratified and selected in two levels. Twenty-one sampling strata were produced after stratifying each region into urban and rural areas. Using probability proportion, 305 enumeration areas (93 from urban and 212 from rural) were selected in the first stage. Newly formed household listing was used in the second stage to choose a set number of thirty households per cluster with an equal probability of systematic selection. Sample allocation was done to verify that survey precision was equivalent across regions. Thirty-five enumeration areas were selected from the three largest regions. Twenty-five enumeration areas were selected from eight regions (including two city administrations). The complete sampling procedure is available in the EDHS 2019 final report (24). In the current study, a total of 1,427 weighted children aged 6–23 month were participated. The spotlight sampling technique for the present study is shown in Figure 1.

### Study variables

The dependent variable is minimum acceptable diet. This study considered different independent variables to identify determinants of level of minimum acceptable diet (Table 1).

**Table 1:** List of independent variables for the assessment of geospatial pattern of level of minimum acceptable diet and its predictors among children in Ethiopia.

Variable	Descriptions (classification)
Sex of the child	Male or female
Age of the child	6–11-month, 12–17-month, 18–23 month
Mothers’ age	15–24, 25–34, 35–49
Residence status	Urban or Rural
Region	Larger central: Tigray, Amhara, Oromia, SNNPR Small peripherals: Benishangul, Gambela, Afar, Somali Metropolis: Harari, Addis Ababa, Dire Dawa (25, 26).
Mothers’ educational status	No education, primary, secondary or higher
Wealth index	Poor, middle, and rich
Marital status	Not married or married
Antenatal	No ANC visit,1–3 or 4 and above
Postnatal care	No or yes
Place of delivery	Home or health institution
Type of birth	Single or twin and above
Family size	2–5, 6–10, >10
Sex of household	Male or female
Birth order	First, 2nd and 3rd, 4th and above
Number of children	One and two, above two
Media access (television)	Yes or no
Media access (radio)	Yes or no

### Definitions

Inadequate minimum acceptable diet (MAD) is defined as the situation when a child is unable to meet the WHO minimum acceptable diet criteria. Minimum acceptable diet is defined as a composite of children fed with a minimum dietary diversity and a minimum meal frequency. Minimum dietary diversity: children age 6–23 months who received a minimum of five out of eight food groups during the previous day. Minimum meal frequency: For breastfed children receiving solid, semisolid, or soft food at least twice a day for infants age 6–8 months aged and at least three times a daily for children age 9–23 months. For non-breastfed children, age 6–23 months aged receiving solid, semisolid, or soft food or milk feeds at least four times a day (27).

### Data processing and analysis

Data were extracted from the EDHS 2019 individual record folder using STATA version 17. Sorting and listing were used to find any missing values. Descriptive statistics were calculated using frequency and percentage. Data were weighted, cleaned, edited, and recoded. STATA version 17 was used for non-spatial analysis. ArcGIS Pro and SatScan version 9.6 were used to map the level of minimum acceptable diet at regional and district levels.

### Spatial autocorrelation analysis

Spatial autocorrelation with Global Moran’s I was computed to determine whether inadequate minimum acceptable diet among children is randomly distributed, clustered, or dispersed. Global Moran’s I value near “0” indicates inadequate minimum acceptable diet is randomly distributed, near “–1” shows dispersed, and close to “+1” indicates clustered. Spatial autocorrelation is declared at a statistically significant Moran’s I *p*-value less than 0.05.

### Hot spot and spatial interpolation analysis

Hot spot analysis was done using Getis-Ord Gi* Spatial Statistics to identify statistically significant spatial clusters of cold spots (low) and hot spots (high) of inadequate minimum acceptable diet. Clusters with high Gi* values are statistically significant hot spots, while clusters with low Gi* values are statistically significant cold spots. Ordinary kriging interpolation was computed to estimate the value of unsampled locations from sampled data points.

### Ordinary least squares and multiscale geographically weighted regression analysis

Ordinary least squares (OLS) regression was used to model the relationship between inadequate minimum acceptable diet and a set of independent variables, using data from a sample of 295 enumeration areas. The outcome variable for the regression model was the weighted inadequate minimum acceptable diet prevalence in each cluster. OLS regression assumes that the relationship between each explanatory variable and the outcome variable is the same throughout the study area (28). However, a constant relationship between the explanatory variables and the outcome variable may not always be valid. Thus, exploring further through geographically weighted regression (GWR) is crucial, which allows the coefficients to vary across the study area.

A newly emerging geographic regression model called MGWR was used to explore geographically varying relationships between inadequate minimum acceptable diet and explanatory variables. Unlike GWR, MGWR not only allows the coefficients to vary over space but also allows the scale to vary across different covariates. MGW considers distinct neighborhoods for each covariate to account for different spatial scales of the relationships between each explanatory variable and the outcome variable (29). MGWR is a powerful tool that can be used to model complex spatial relationships between variables. Variance inflation factor was calculated to check the exitances of multicollinearity between variables. A VIF above 4 indicates multicollinearity might exist (30, 31). Akaike information criterion (AIC) and adjusted *R*^2^ values were computed to select the appropriate model. The model with the lowest AICc and higher adjusted *R*^2^ was declared the best-fitted model (32). In all statistical analyses, the statistical significance was considered at *p*-value <0.05.

## Results

### Characteristics of study participants

An analysis of 1,427 children aged 6–23 months was conducted to determine geospatial pattern and predictors of level of minimum acceptable diet in Ethiopia. Form 697(48.84%) children of Mothers’ with no education, the majority (46.95%) of children did not achieve the minimum acceptable diet. Regarding the wealth index, of 661 (46.32%) children in the poor household wealth category, only 23 (1.61%) get the minimum acceptable diet. Additionally, in households with three or more children, only 3.85% of children met the minimum acceptable diet (Table 2).

**Table 2:** Individual level characteristics of study participants EDHS 2019, (weighted *n* = 1,427).

Variables	Categories	Minimum acceptable diet		Total (weighted), (%)
		Achieved (%)	Not achieved (%)	
Sex of the child	Male	74(5.19%)	653(45.76%)	727(50.95%)
	Female	75(5.26%)	625(43.80%)	700(49.05%)
Age of the child	6–11 months	33(2.31%)	458(32.10%)	491(34.41%)
	12–17 months	64(4.48%)	469(32.87%)	533(37.35%)
	18–23 months	52(3.64%)	351(24.60%)	403(28.24%)
Age of the Mothers’	15–24	43(3.01%)	398(27.89%)	441(30.90)
	25–34	88(6.17%)	673(47.16%)	761(53.33%)
	35–49	18(1.26%)	207(14.51%)	225(15.77%)
Place of residence	Urban	298(20.88%)	76(5.33%)	374(26.21%)
	Rural	980(68.68%)	73(5.12%)	1,053(73.79%)
Region	Large central	536(37.56%)	64(4.48%)	600(42.05%)
	Small peripheral	491(34.41%)	29(2.03%)	520(36.44%)
	Metropolitan	251(17.59%)	56(3.92%)	307(21.51%)
Educational status	No education	27(1.89%)	670(46.95%)	697(48.84%)
	Primary education	63(4.41%)	434(30.41%)	497(34.83%)
	Secondary education	29(2.03%)	103(7.22%)	132(8.25%)
	Higher education	30(2.10%)	71(4.98%)	101(7.08%)
Marital status	Married	137(9.60%)	1,198(83.95%)	1,335(93.55%)
	Not married	12(0.84%)	80(5.61%)	92(6.45%)
Wealth index level	Poor	23(1.61%)	638(44.71%)	661(46.32%)
	Middle	26(1.82%)	187(13.1%)	213(14.93%)
	Rich	100(7.01%)	453(31.74%)	553(38.75%)
Family size	2–5	89(6.24%)	627(43.94%)	716(2018%)
	6–10	57(3.99%)	606(42.47)	663(46.46%)
	>10	3(0.21%)	45(3.15%)	48(3.36%)
Sex of the household head	Male	118(8.27%)	991(69.45%)	1,109(77.72%)
	Female	31(2.17%)	287(20.11%)	318(22.28%)
Birth order	First	57(3.99%)	265(18.57%)	322(22.56%)
	2nd and 3rd	53(3.71%)	478(33.5%)	531(37.21%)
	4th and above	39(2.73%)	535(37.49%)	574(40.22%)
Number of children	One and two	94(56.59%)	564(39.52%)	658(46.11%)
	Three and above	55(3.85%)	714(50.04%)	769(53.89%)
Media access (television)	Yes	79(5.54%)	1,054(73.86%)	1,133(79.40%)
	No	70(4.91%)	224(15.70%)	294(20.60%)
Media access (radio)	Yes	84(5.89%)	963(67.48%)	1,047(73.37%)
	No	65(4.56%)	315(22.07%)	380(26.63%)
Place of delivery	Home	22(1.54%)	590(41.35%)	612(42.89%)
	Health institution	127(8.90%)	688(48.21%)	815(57.11%)
ANC	No ANC visit	16(1.12%)	345(24.18%)	361(25.30%)
	1–3 visit	32(2.24%)	429(30.06%)	461(32.31%)
	4 and above visit	101(7.08%)	504(35.32%)	605(42.40%)
PNC checkup within 2 months	No	109(7.64%)	1,133(79.40%)	1,242(87.04%)
	Yes	40(2.80%)	145(10.16%)	185(12.96%)

ANC stands for antenatal care, and PNC stands for postnatal care.

In this study, the majority of the children (89.56%, 95CI, 87.85–91.10%) did not achieve the minimum acceptable diet according to the WHO criteria.

### Spatial and incremental autocorrelation

**Figure 2:**
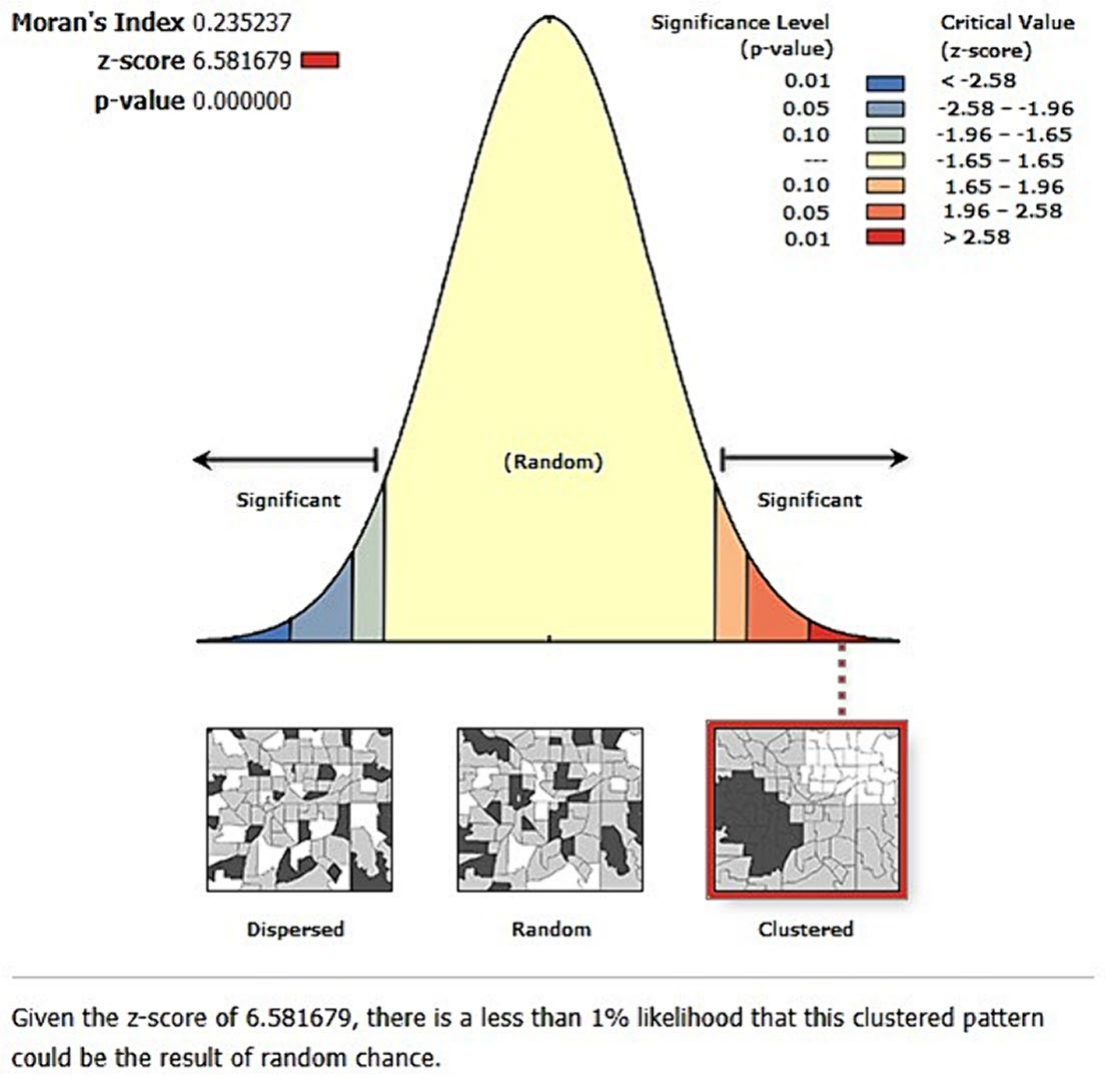
Spatial autocorrelation analysis of geospatial pattern of level of minimum acceptable diet among children in Ethiopia, EDHS 2019, (weighted *n* = 1,427).

**Figure 3:**
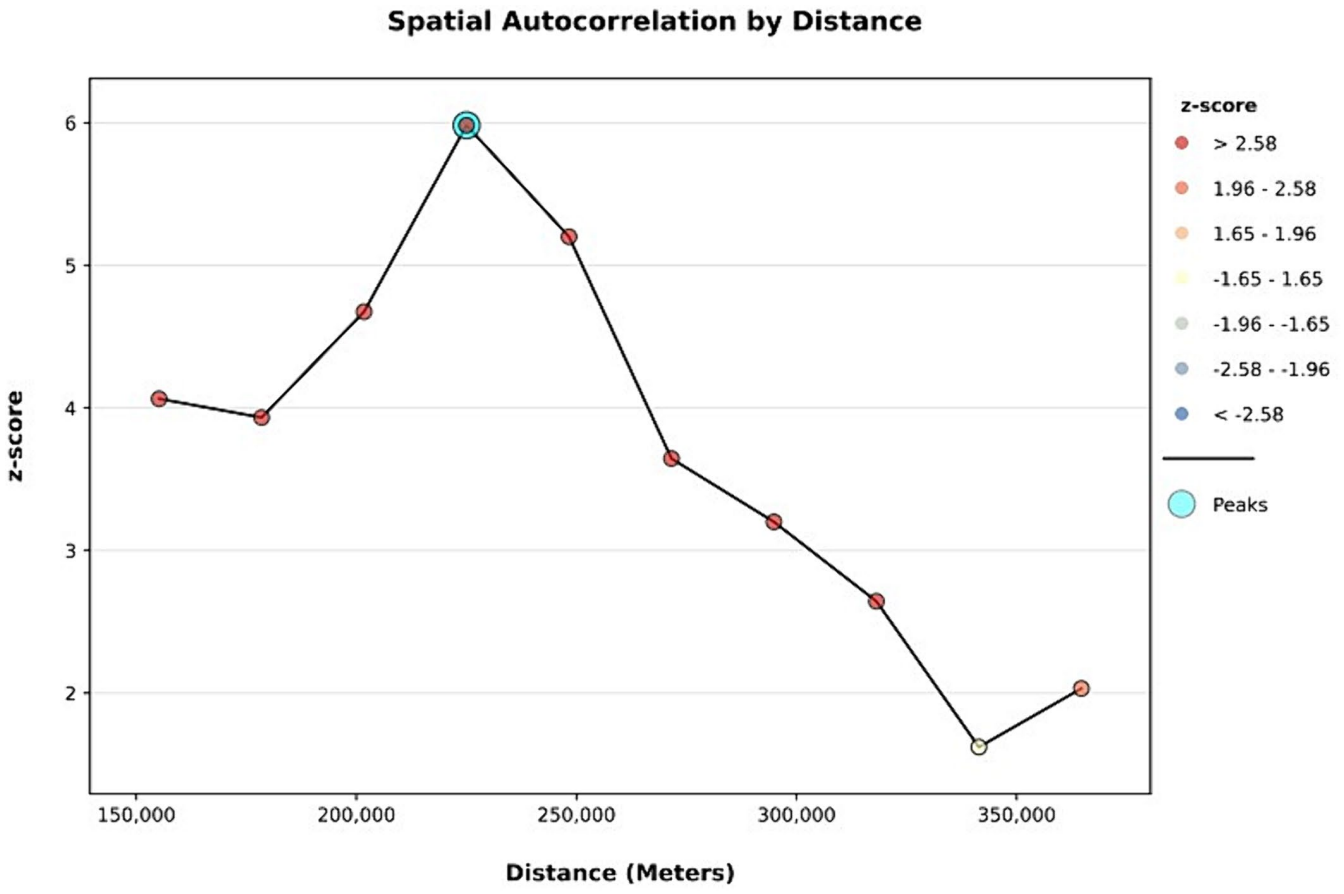
Incremental autocorrelation analysis of geospatial pattern of level of minimum acceptable diet among children in Ethiopia, EDHS 2019, (weighted *n* = 1,427).

The spatial distribution of inadequate minimum acceptable diet among children aged 6–23 months in Ethiopia is clustered with Global Moran’s *I* value of 0.235, *p*-value <0.001, and *Z*-score of 6.58. Thus, a minimum acceptable diet has a spatial dependency. In addition, the likelihood of this clustered pattern because of random chance is less than 1% (Figure 2). The line graph of incremental autocorrelation shows the minimum and the maximum distance band. The minimum distance at the beginning was 155193.00 m (*Z*-score = 4.06, *p*-value <0.001), whereas the first maximum peak was 225028.86 m (*Z*-score = 5.98, *p*-value <0.001) (Figure 3).

### Hotspot and cold spot analysis

**Figure 4:**
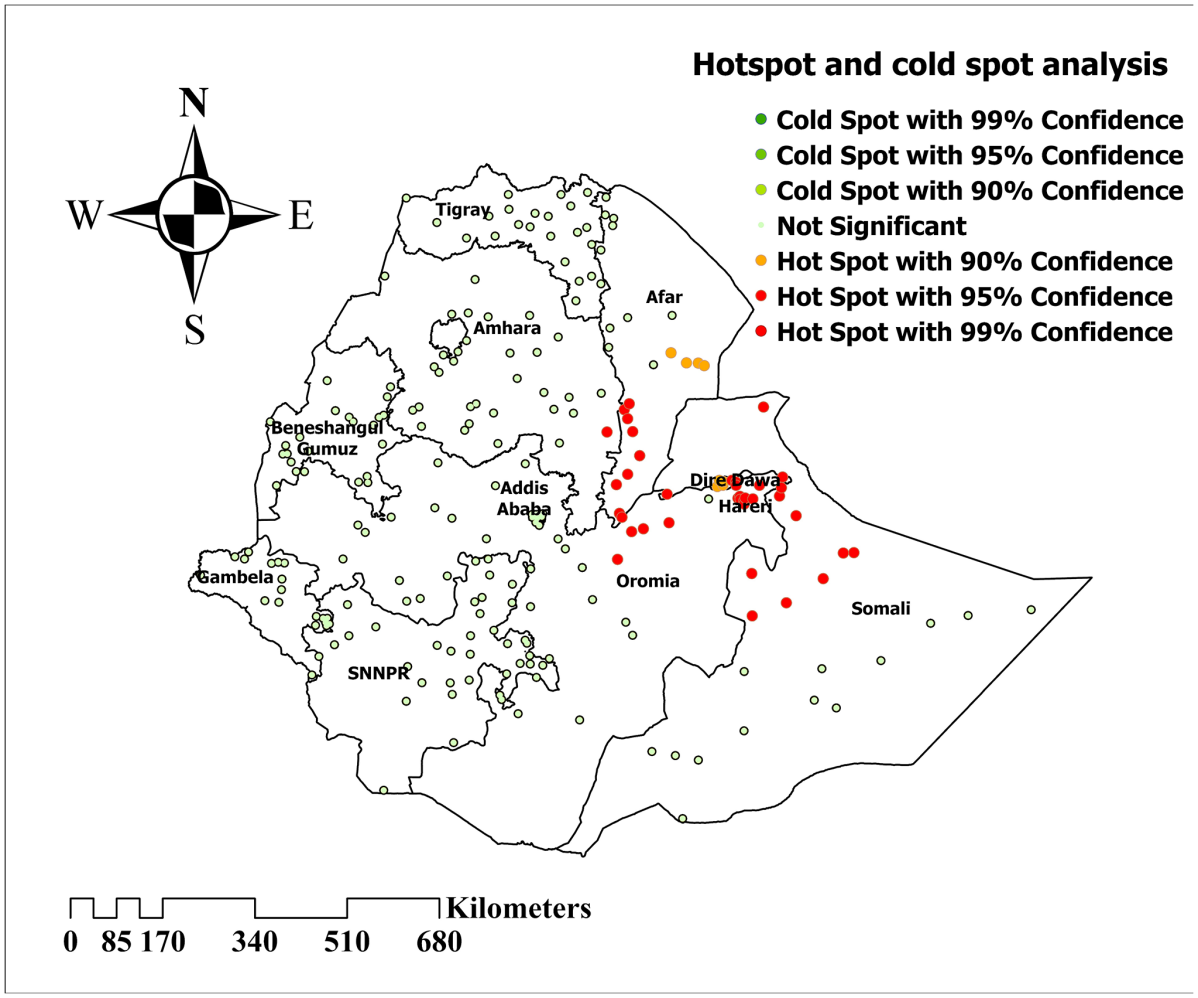
Hotspot and cold spot analysis of inadequate minimum acceptable diet among children in Ethiopia, EDHS 2019, (weighted *n* = 1,427).

Hotspot and cold spot analysis was computed to identify areas with a high and low prevalence of inadequate minimum acceptable diet. Thus, significant clustering of inadequate minimum acceptable diet is detected in Afar and Somali regions (Figure 4).

### Spatial interpolation

**Figure 5:**
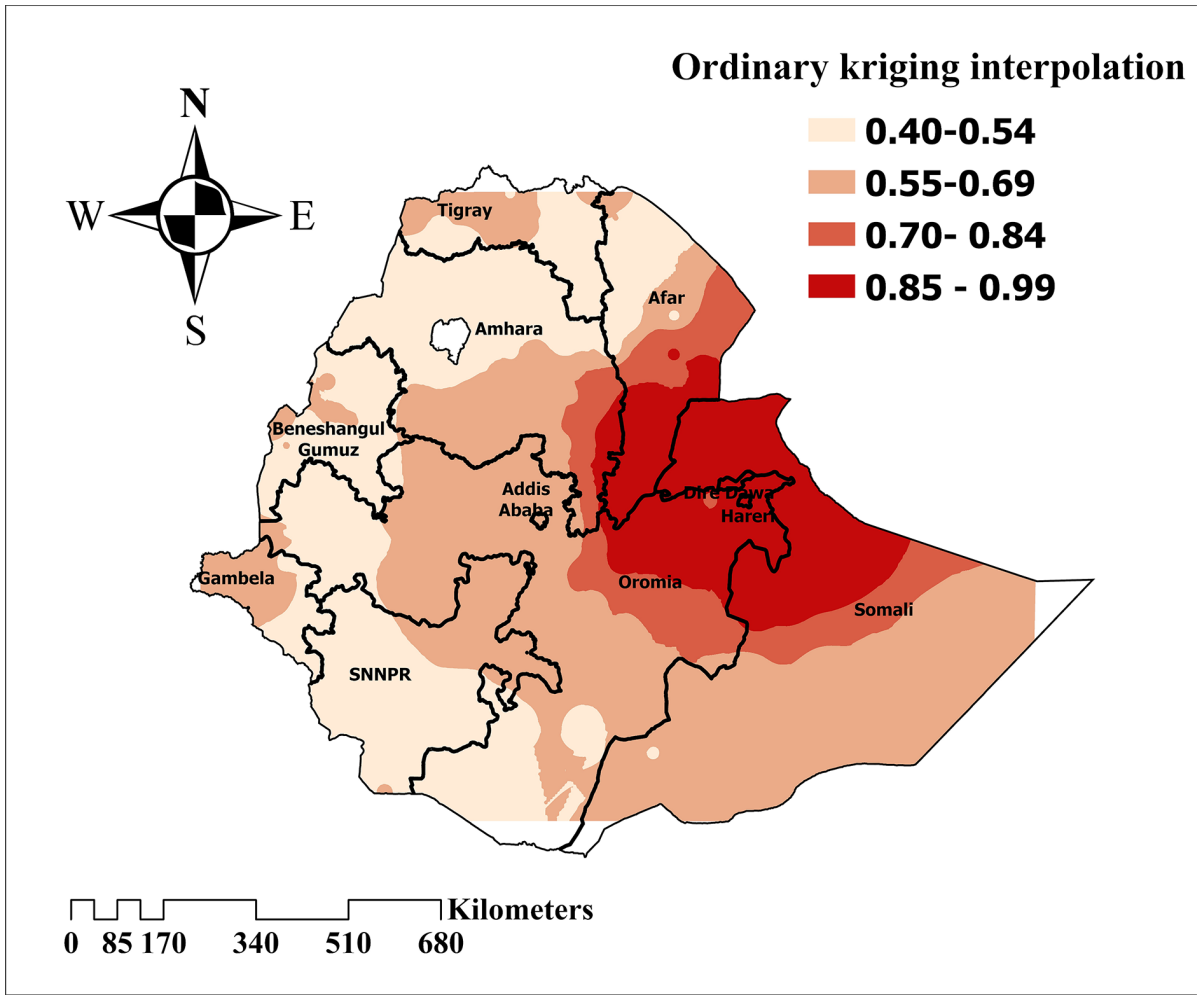
Ordinary kriging interpolation to predict the prevalence of inadequate minimum acceptable diet among children in Ethiopia, EDHS 2019, (weighted *n* = 1,427).

Ordinary kriging interpolation was computed to predict the inadequate minimum acceptable diet distribution among children in Ethiopia. Thus, the highest predictive prevalence of inadequate minimum acceptable diet increased in the Somali, Afar, and Amhara regions. Meanwhile, low prediction was found in the remaining areas of Ethiopia (Figure 5).

### Satscan analysis

**Figure 6:**
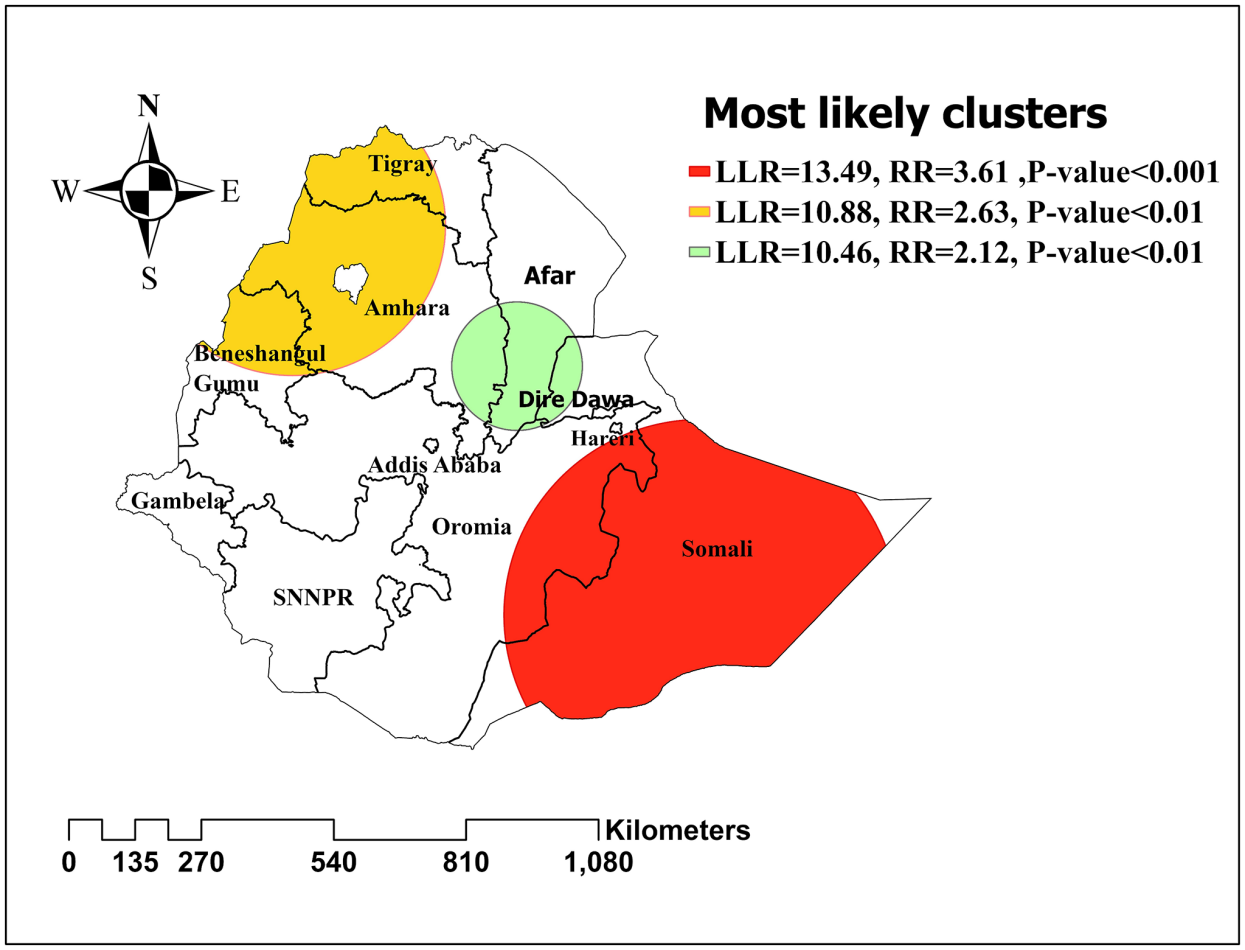
Spatial clustering of high and low rate of inadequate minimum acceptable diet among children in Ethiopia, EDHS 2019, (weighted *n* = 1,427).

Purely spatial analysis using the Bernoulli model was done to identify clusters with high or low inadequate minimum acceptable diet rates. The primary significant sat scan was identified in Eastern Ethiopia (Somali) at 5.856584 N, 43.726016 E/geographic location (radius of 399.27 km) with a relative risk of 3.61 (*p*-value ≤ 0.001) and Log likelihood ratio (LLR) of 13.49. In that highly cluttered area, there was over three and a half times the risk of inadequate minimum acceptable diet. The prevalence of inadequate minimum acceptable diet was higher with the circle hole than the outside (Figure 6).

### Ordinary least squares (OLS) model results

An ordinary least squares model was computed to identify spatial predictors of inadequate minimum acceptable diet. Mothers’ with no formal education, family size above five, no media access, no ANC utilization, no PNC checkup after delivery, and poor wealth index were found to have relationships with inadequate minimum acceptable diet among children in Ethiopia. Multicollinearity was checked by computing the Variance inflation factor (VIF). The maximum and the minimum VIF were 3.609 and 2.161, respectively. Thus, there is no significant multicollinearity between explanatory variables. Joint *F*-Statistic and Joint Wald Statistic result (*p*-value <0.05) shows that the model is statistically significant.

On the other hand, the Jarque-Bera Statistic result (*p*-value >0.05) explained that the OLS is free from bias. Furthermore, Koenker’s (BP) Statistic was found to be statistically significant (*p*-value <0.05). Therefore, there is a possibility of heteroscedasticity and/or non-stationarity. Thus, the model is a good candidate for further multiscale geographically weighted regression (GWR) analysis (Table 3).

**Table 3:** Summary Ordinary Least Squares (OLS) regression result.

Variables	Coefficients	Robust-statistics	Robust probability	VIF
Intercept	0.181	1.885	0.060	
Rural residence	0.064	1.819	0.069	2.899
Mothers’ with no formal education	0.118	3.351	0.000^***^	2.768
Poor Household Wealth index	0.007	0.165	0.868	3.609
Family size above five	0.152	3.794	0.000^***^	2.161
No media access	0.115	2.306	0.021^*^	3.164
Home delivery	0.072	1.467	0.143	3.301
No ANC utilization	0.144	2.496	0.013^*^	3.341
No PNC checkup	0.069	3.12	0.000^***^	2.963
Ordinary least square regression diagnostics				
Number of observations	295	AIC		777.009
Multiple *R*-squared	0.876	Adjusted *R*-Squared		0.873
Joint *F*-Statistic	254.129	Prob(>F), (8,286) DF		0.000
Joint Wald statistic	3214.006	Prob(>chi-squared), (10) DF		0.000
Koenker (BP) statistic	18.700	Prob(>chi-squared), (10) DF		0.016
Jarque–Bera statistic	13.404	Prob(>chi-squared), (2) DF		0.122

ANC stands for antenatal care, and PNC stands for postnatal care. Determinants at *p*-value < 0.05 = *, <0.01 = ** and <0.001 = ***.

### Geographically weighted regression analysis

The model comparison was done by comparing AIC and R-squared values for each model. A model with a small AIC and a high value of *R*^2^ was considered the best model. Thus, the MGWR model was favorable, with AIC and *R*^2^ values of 256.388 and 0.891, respectively. The MGWR analysis revealed that mothers’ with no formal education, family size above five, no media access, home delivery, and no PNC checkup after delivery were found to have a positive relationship with inadequate minimum acceptable diet (Table 4).

**Table 4:** Summary of geographically weighted regression analysis result and model comparison.

Variables	Mean	Minimum	Maximum	*P*-value
Intercept	0.014	0.009	0.061	
Rural residence	0.087	0.008	0.158	0.081
Mothers’ with no formal education	0.043	0.078	0.113	0.000^***^
Poor Wealth index	0.023	0.017	0.026	0.875
Family size above five	0.076	0.066	0.098	0.005^**^
No media access	0.059	0.041	0.076	0.030^*^
Home delivery	0.078	0.066	0.158	0.002^**^
No ANC utilization	0.113	0.916	0.157	0.056
No PNC checkup	0.131	0.092	0.213	0.000^***^
Model comparison (OLS vs. GWR vs. MGWR)				
Parameters	OLS	GWR	MGWR	
AIC	777.009	260.548	256.388	
*R*-squared	0.876	0.880	0.891	
Adjusted *R*-squared	0.873	0.872	0.890	

Determinants at *p*-value < 0.05 = *, <0.01 = ** and <0.001 = ***.

**Figure 7:**
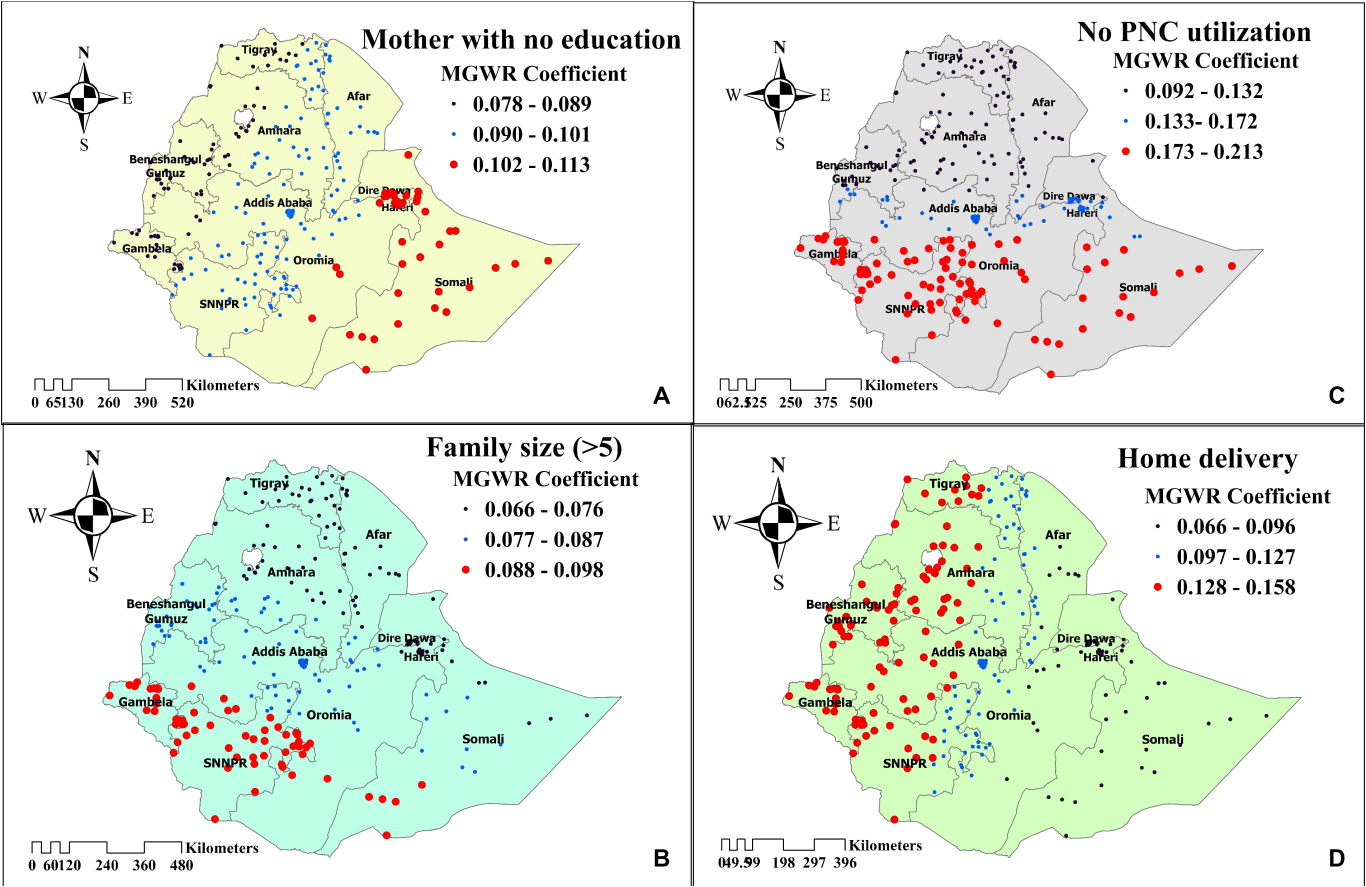
The spatial mapping of geographically weighted regression coefficients by mothers’ with no education **(A)**, family size (>5) **(B)**, no PNC utilization **(C)**, and home delivery **(D)** to predict the hotspot of inadequate minimum acceptable diet among children in Ethiopia.

Analyzing the MGWR graph revealed that a 1% increase in the number of mothers’ with no formal education was associated with a 4.3% higher likelihood of not achieving the minimum acceptable diet, particularly in the eastern part of Ethiopia (Somali regions) (Mean = 0.043, *p*-value = 0.000). In southern Ethiopia (Gambela and SNNPR regions), household family size was the main factor influencing the likelihood of not achieving the minimum acceptable diet. As the number of households with a family size above five increased, the probability of not achieving the minimum acceptable diet rose by 7.6% (Mean = 0.076, *p*-value = 0.005). Furthermore, a decrease in the number of mothers who utilized postnatal care (PNC) was associated with an 13.1% higher chance of not achieving the minimum acceptable diet (Mean = 0.131, *p*-value = 0.000), primarily in the southwestern and eastern parts of Ethiopia. Additionally, with other factors held constant, children from communities with high home delivery rates were 7.8% more likely to have an inadequate minimum acceptable diet in the northern and western parts of Ethiopia (Mean = 0.078, *p*-value = 0.002). In areas with inadequate media access, the likelihood of not achieving the minimum acceptable diet increases by 5.9% (Mean = 0.059, *p*-value = 0.030) (Figure 7).

## Discussion

This study aimed to determine the geospatial pattern of level of minimum acceptable diet and its predictors among children aged 6–23 months in Ethiopia. The final analysis revealed that 89.56% of the children were unable to achieve the minimum acceptable diet. Besides, The MGWR analysis revealed that mother with no formal education, family size above five, no media access, home delivery, and no PNC checkup after delivery were found to be the determinates of level of minimum acceptable diet.

In this study majority of the child (89.56%, 95CI: 87.85–91.10%) failed to achieve the minimum acceptable diet recommended by the WHO. This finding is higher than the study done in in Nepal (71%) (33), Indonesia (52.3%) (34), and Congo (67%) (10). In contrast, the finding is lower than the study done in India (91.6%) (11), Uganda (94.7%) (35), and Guinea (96.9%) (36). However, the finding is consistent with the study done in East Africa (88.5%) (37). These observed differences could be due to various factors, such as regional variations, population growth rates, and socioeconomic disparities between the countries being compared (38). Additionally, cultural backgrounds and individual perceptions of a balanced diet can significantly impact dietary choices (14). Governmental initiatives implementing national nutrition programs focused on proper feeding practices could also play a significant role in improving child feeding practices, thereby contributing to achieving a MAD (39). Moreover, the implementation of the Baduta program by the Global Alliance for Improved Nutrition (GAIN) has contributed to improving the achievement of an acceptable minimum diet in Indonesia (40). To address this issue effectively, tailored strategies should be developed, taking into account the variations observed across different regions. Continuous monitoring and evaluation of nutritional programs are crucial for assessing their impact and making necessary adjustments. Furthermore, translating research findings into actionable insights through various dissemination channels is key for informing policy and practice, ultimately leading to improved child nutrition outcomes.

The prevalence of inadequate minimum acceptable diet among children in Ethiopia exhibits substantial geographical variation, ranging from 40 to 99%. This finding aligns with a similar study conducted in India, confirming the existence of regional disparities in child dietary practices (41). This spatial dependency of child feeding practices stems from geographical variations in cultural norms, socioeconomic status, educational attainment, and maternal healthcare utilization, all of which influence the likelihood of children achieving a minimum acceptable diet (42, 43).

Children whose mothers have no formal education were unable to achieve the minimum acceptable diet in high hotspot areas (Somali regions). Several other studies also report a positive association between mother’s educational level and meeting MAD (15, 16, 44). In high hotspot areas like the Somali regions, which have the highest women illiteracy rate (73.3%) among Ethiopia’s regions, the absence of formal education among mothers correlates with challenges in providing children with minimum acceptable diets. Firstly, socioeconomic status plays a crucial role, as mothers with no formal education often hail from lower-income backgrounds where access to nutritious food is limited due to financial constraints. In such circumstances, families may prioritize cheaper but less nutritious food options, compromising the quality of their children’s diets. Additionally, the lack of formal education may result in a deficit of nutritional knowledge among mothers, hindering their ability to make informed decisions regarding their children’s dietary needs. Without the understanding of the importance of a balanced diet and how to achieve it, mothers may unintentionally provide inadequate nutrition to their children. Moreover, societal factors such as limited access to healthcare and community resources can further exacerbate the challenge of attaining a minimum acceptable diet for these children.

In households with a family size above five, children fail to achieve the minimum acceptable diet in southern Ethiopia. Likewise, in another study, an increase in the number of children reduces the likelihood of attaining MAD (45). In southern Ethiopia, myths and misconceptions regarding family planning (46) might contribute to an increase in family size, leading to challenges in achieving the minimum acceptable diet. Moreover, factors such as unmet need for family planning in this areas (47) may also play a role. Addressing the nutritional challenges associated with family size in southern Ethiopia requires interventions that promote reproductive health education, improve access to family planning resources, and provide economic empowerment opportunities for women. These measures can help families make informed decisions about family size while ensuring the well-being of their children.

In regions with limited access to media resources, children are unable to achieve the recommended minimum acceptable diet. This is in accordance with the findings of a previous study (48), which indicated a similar trend. The lack of access to media sources among parents hinders their ability to gain adequate knowledge about food diversification and maintaining proper meal frequency, which are crucial factors in achieving the recommended minimum acceptable diet for their children. Thus, improving media access to disseminate essential nutrition-related information will improve child feeding practices.

In western Ethiopia, the practice of home delivery is the main factor hindering the achievement of the minimum acceptable diet for children. This finding aligns with the study conducted in Nepal (49). Home deliveries often lack the involvement of skilled healthcare providers who can offer guidance on proper child feeding practices, including the importance of timely introduction of complementary foods, maintaining dietary diversity, and ensuring adequate meal frequency. In western Ethiopia, it is evident that the prevalence of home delivery is high, reaching up to 80% (50). Consequently, children in households that rely on home deliveries may be more susceptible to malnutrition and its associated health complications. This underscores the need for targeted interventions to address the challenges associated with home delivery and promote optimal child-feeding practices in regions where home delivery is widespread. Improve access to healthcare facilities by investing in infrastructure and maternal health services is also crucial.

In southern and eastern Ethiopia, the failure to attend postnatal care (PNC) checkups significantly impacts children’s ability to achieve the minimum acceptable diet. These regions often face challenges such as remote locations, limited healthcare infrastructure, and cultural beliefs that may deter mothers’ from seeking healthcare services (51, 52). Consistent findings was obtained from other studies (14, 53). Mothers who forgo PNC checkups miss essential opportunities to receive comprehensive counseling and support on optimal infant and young child feeding (IYCF) practices. These checkups provide a critical platform for healthcare providers to educate mothers’ on various aspects of IYCF, including dietary diversity and meal frequency. Implementing programs that enhance PNC utilization is therefore crucial. Besides, including essential child nutritional education and demonstration in PNC checkup will be crucial to achieve MAD.

The use of nationally representative data enhances this study’s generalizability and statistical power, allowing for the application of its findings on a national scale. Additionally, the employment of SaTScan and spatial distribution analysis provides valuable insights into the geographical distribution of inadequate minimum acceptable diet. However, the study’s reliance on secondary data limits its ability to delve into the underlying causes of this phenomenon. The data may not encompass all the factors contributing to inadequate minimum acceptable diet. Furthermore, the cross-sectional nature of the data hinders the establishment of causal relationships between the explanatory variables and minimum acceptable diet. Moreover, the lack of longitudinal data prevents the study from tracking changes in achieving a minimum acceptable diet over time and identifying potential risk factors.

## Conclusion

In Ethiopia, 89.56% of children were unable to meet the minimum acceptable diet, surpassing the global average of 84% (54). This alarming statistic underscores the urgent need for targeted interventions, particularly in high-risk areas like the Afar and Somali regions. Additionally, allocating resources to these areas is crucial to expedite progress toward enhancing child feeding practices. Mothers’ with no formal education, family size exceeding five, no media access, home deliveries, and the absence of postnatal care (PNC) checkups were found to be the determinates of inadequate minimum acceptable. Community-based initiatives to promote women’s education have a pivotal role in addressing the problem. Programs that encourage institutional deliveries and enhance the utilization of postnatal care services are also highly recommended. Moreover, managing family size can significantly contribute to achieving the minimum acceptable diet for children. Overall, addressing those contributing factors in identified geographical locations holds immense potential to improve child nutrition and foster optimal child growth.

## Data availability statement

The datasets presented in this study can be found in online repositories. The names of the repository/repositories and accession number(s) can be found at: https://dhsprogram.com/.

## Ethics statement

Ethical approval was not required for the studies involving humans because The EDHS 2019 was ethically reviewed by the National Research Ethics Review Committee (NRERC) of the Ethiopian Ministry of Science and Technology. As described in the survey final report, involvement in the survey program was voluntary, and verbal agreement (informed consent) was also taken (23). The studies were conducted in accordance with the local legislation and institutional requirements.

## Author contributions

TK: Conceptualization, Formal analysis, Methodology, Software, Writing – original draft, Writing – review & editing. BA: Methodology, Resources, Software, Validation, Visualization, Writing – review & editing. BT: Conceptualization, Data curation, Funding acquisition, Validation, Visualization, Writing – review & editing. RH: Funding acquisition, Investigation, Methodology, Project administration, Software, Supervision, Validation, Writing – original draft.
